# Caregiver Needs and Resources—2023 Summer Styles Survey, United States

**DOI:** 10.1002/alz.089072

**Published:** 2025-01-09

**Authors:** Jenny A Walker, Greta Kilmer

**Affiliations:** ^1^ Centers for Disease Control and Prevention, Atlanta, GA USA

## Abstract

**Background:**

The United States population is aging, with more individuals requiring care—a shift that has made addressing the needs of the nation’s caregiver population a public health priority. Understanding the resources caregivers use and need can inform those public health efforts.

**Method:**

We examined 2023 Summer Styles survey data addressing caregiver services and support among U.S. adult caregivers (n = 437). Participants responded to questions about their need for and utilization of seven resources: out‐of‐home services, in‐home services, support groups or classes, care recipient food and nutrition support services, care recipient transportation services, care recipient financial planning services, and caregiver training opportunities. We further analyzed results from caregivers reporting that it was somewhat or very difficult to afford household expenses (n = 90), and those who care for a person with dementia or another cognitive impairment (n = 96). All percentages were weighted to match the U.S. Current Population Survey proportions.

**Result:**

Overall, 45.5% of the surveyed caregivers indicated that they have utilized or would utilize one or more resource type. In‐home services were the most common (33.1%), followed by transportation services (26.6%) and care recipient food and nutrition services (25.2%). Care recipient financial planning resources were the least common (13.0%). About one third (34.2%) of caregivers indicated they would use at least one of the seven resources, but that it was unavailable or not known to be available to them. Among caregivers who struggle with household expenses, transportation services were the most frequently identified (29.3%) resource they would use if available. Among those who care for a person with dementia or another cognitive impairment, in‐home services was the most frequently identified (21.8%) resource they would use if available.

**Conclusion:**

These results suggest that caregiver resource use and need varies qualitatively based on financial limitations and the diagnosis of the care recipients. Additionally, over a third of caregiver respondents indicated that they would use a service or tool, but it is currently not available or known to be available to them. These results can help inform public health decision‐making in addressing the needs of the nation’s caregivers.

